# Determinants of participation in worksite health promotion programmes: a systematic review

**DOI:** 10.1186/1479-5868-6-26

**Published:** 2009-05-20

**Authors:** Suzan JW Robroek, Frank J van Lenthe, Pepijn van Empelen, Alex Burdorf

**Affiliations:** 1 Department of Public Health, Erasmus MC, University Medical Center Rotterdam, PO Box 2040, 3000 CA Rotterdam, the Netherlands

## Abstract

**Background:**

The workplace has been identified as a promising setting for health promotion, and many worksite health promotion programmes have been implemented in the past years. Research has mainly focused on the effectiveness of these interventions. For implementation of interventions at a large scale however, information about (determinants of) participation in these programmes is essential. This systematic review investigates initial participation in worksite health promotion programmes, the underlying determinants of participation, and programme characteristics influencing participation levels.

**Methods:**

Studies on characteristics of participants and non-participants in worksite health promotion programmes aimed at physical activity and/or nutrition published from 1988 to 2007 were identified through a structured search in PubMed and Web of Science. Studies were included if a primary preventive worksite health promotion programme on PA and/or nutrition was described, and if quantitative information was present on determinants of participation.

**Results:**

In total, 23 studies were included with 10 studies on educational or counselling programmes, 6 fitness centre interventions, and 7 studies examining determinants of participation in multi-component programmes. Participation levels varied from 10% to 64%, with a median of 33% (95% CI 25–42%). In general, female workers had a higher participation than men (OR = 1.67; 95% CI 1.25–2.27]), but this difference was not observed for interventions consisting of access to fitness centre programmes. For the other demographic, health- and work-related characteristics no consistent effect on participation was found. Pooling of studies showed a higher participation level when an incentive was offered, when the programme consisted of multiple components, or when the programme was aimed at multiple behaviours.

**Conclusion:**

In this systematic review, participation levels in health promotion interventions at the workplace were typically below 50%. Few studies evaluated the influence of health, lifestyle and work-related factors on participation, which hampers the insight in the underlying determinants of initial participation in worksite health promotion. Nevertheless, the present review does provide some strategies that can be adopted in order to increase participation levels. In addition, the review highlights that further insight is essential to develop intervention programmes with the ability to reach many employees, including those who need it most and to increase the generalizability across all workers.

## Background

The imbalance between physical activity (PA) and nutrition is an important cause of overweight and obesity, which in turn are important risk factors for cardiovascular diseases (CVD), and other chronic diseases [1]. The World Health Organization reported that, globally, there are more than one billion overweight adults and at least 400 million obese adults [2]. In the primary prevention of obesity, a large variety of health promotion programmes are offered.

In the past decades the workplace has been identified as an important setting for health promotion, since it offers an efficient structure to reach large groups, and makes use of a natural social network [3,4]. Research has thus far mainly focused on the effectiveness of these interventions. There are, however, several reasons to also investigate participation in health promotion programmes at the workplace. Firstly, the effectiveness of a worksite health promotion programme (WHPP) will be influenced by the characteristics of the target population and the proportion of the population that enrols in the offered intervention. As such, differences in participation levels may partly explain the large differences in effectiveness of WHPPs observed [3,5,6]. Secondly, WHPPs have to deal with variable and often low participation levels [7]. This may hamper the external validity of the findings, particularly when selective groups of individuals participate in the programmes. Earlier studies addressing participation in worksite health promotion [7-10] presented participation levels varying from 8% to 97% [7]. In a review, Glasgow and colleagues (1993) reported that men, blue-collar employees, and smokers appeared less likely to participate [9]. In accordance with these findings, Dobbins and colleagues (1998) found a higher attendance in an at-work health risk assessment for women and those of higher occupational class. A lower participation was found among current or past smokers, but no differences were found for alcohol consumption, physical activity, and nutrition [8]. Thirdly, low participation will result in low cost-effectiveness.

Since the last systematic review on participation in WHPPs in 1993 [9], numerous worksite programmes aiming at physical activity, nutrition and overweight have been evaluated for their cost-effectiveness. Knowledge about programme characteristics that contribute to participation is required to increase the cost-effectiveness of the interventions, which may be crucial for companies implementing the programmes. In order to update and extent previous findings it is important to investigate (1) who are reached by means of WHPPs on physical activity and nutrition, and (2) when participation is more likely. Hence, we conducted a systematic review with the aims 1) to describe participation levels in WHPPs, 2) to evaluate underlying individual, health- and work-related determinants of participation, and 3) to analyse programme characteristics that influence participation levels.

## Methods

### Identification of the studies

Relevant articles were identified by means of a computerized search in the bibliographic databases PubMed and Web of Science from 1988 up to December 2007. The following combination of Mesh-terms and keywords was used: (Workplace OR employee* OR worker*) AND (exercise OR fitness OR (physical activity) OR sport OR nutrition OR fat OR fruit* OR vegetable*) AND (intervention OR program*) AND (participa* OR response OR respondent*). For the literature search in Web of Science the Mesh terms were converted to keywords. For inclusion articles had to fulfil the following criteria: (1) the article described a WHPP on physical activity and/or nutrition as primary preventive intervention (primary prevention has been defined as the promotion of health by personal and community-wide efforts [11]) (2) a quantitative description of determinants of initial participation at the start of the programme was given, (3) the association between demographic, health-related, or work-related determinants and participation was expressed in a quantitative measure, such as an odds ratio, or sufficiently raw data were provided to calculate these associations, and (4) the article was written in English.

### Selection

**Figure 1:**
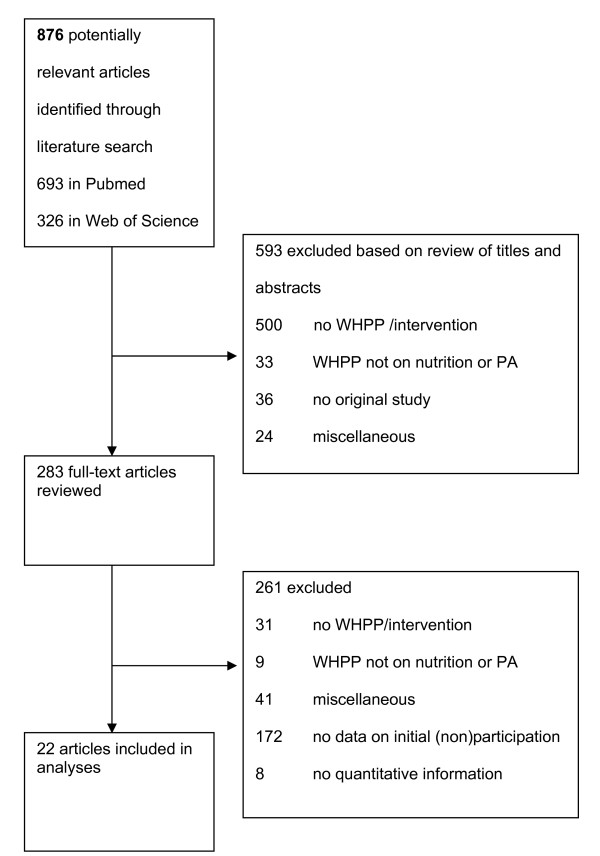
**Flow chart**.

The first author (SR) performed the initial selection of abstracts in the literature search. In case of doubt, the last author (AB) was consulted. Figure 1 shows the flow of the articles throughout the inclusion process. Based on title and abstract, 593 out of 876 articles were discarded because 500 abstracts (57%) did not describe a WHPP, 33 abstracts (4%) were on a WHPP other than nutrition or physical activity, and another 36 abstracts (4%) were no original studies. Finally, 24 abstracts (3%) were excluded for a variety of reasons, such as describing characteristics of worksites that offer a WHPP instead of employees that do or do not participate (n = 7), no primary prevention (n = 4), and willingness to participate instead of actual participation (n = 2).

In total, 283 articles were retrieved for full review, of which 31 out of 261 (12%) were excluded due to not describing a WHPP, 9 (3%) because they did not describe a programme on nutrition or physical activity, and 41 articles (16%) were excluded for a variety of reasons. Of the remaining 180 articles describing a WHPP on nutrition or PA, 172 (96%) did not include any information on characteristics of non-participation and 8 studies (4%) did not include any quantitative information on these characteristics. Finally, 22 (9%) publications met our inclusion criteria.

### Data extraction

A data form was used to extract information on the number of participants, the target population, demographic (e.g. sex, marital status) as well as health- (e.g. physical activity, weight) and work-related (e.g. job type, company size) determinants of participation. Finally, programme characteristics as the availability of incentives, the requirement of paying a fee to participate, the programme type and the targeted behaviour were obtained. The first author (SR) performed the data extraction and the last author (AB) verified all extracted data. In case of doubt, data were discussed until agreement was reached.

After the data extraction, programmes were divided in three groups: (1) programmes with a fitness centre or exercise programme as main component, (2) with education or counselling as main component, (3) and multi-component programmes. One study evaluated a fitness centre programme next to a multi-component programme, and described the determinants of participation in both programmes separately [12]. The determinants of this study were considered separately for both programmes, resulting in 22 publications describing 23 studies.

### Data analysis

The first step in the data analysis was to express participation levels as a proportion of the number of eligible participants. Subsequently, the analysis focused on measures of association between determinants of participation and participation levels. In case no measures of association were included in the original article, available raw data in a 2 × 2 table were used to calculate an odds ratio and 95% confidence intervals for dichotomous or categorical measures, with odds ratios above and below 1 representing respectively higher and lower participation. A pooled odds ratio was calculated using a random effects model due to observed heterogeneity between studies. For continuous measures, the difference between means (Δ) among participants and non-participants was calculated and a Cohen's d value was calculated reflecting the standardized difference between means. A d-value of 0.2 was considered to represent a small difference, 0.5 a medium difference, and 0.8 a large difference. The influence of programme characteristics on participation level was analysed by a meta-analytical approach, pooling the participation numbers and total population numbers for the relevant programme characteristics.

## Results

Determinants of participation were reported in 10 studies with education or counselling as main component [13-22], 6 studies on the introduction of a fitness centre or exercise facilities [12,23-27], and 7 studies describing a multi-component programme [12,28-33] (Tables 1, 2, 3). All 23 studies reported demographic factors [12-33], 11 (48%) health-related aspects [12,13,17,21,23-26,31,33], and 7 (30%) work-related determinants [14,17,18,22,29-31]. The participation levels ranged from 10% to 64% [12], with a median of 33% (95% CI: 25%–42%).

**Table 1:** Participation levels and determinants of participation in educational or counselling worksite health promotion programmes

**Study**	**Study** **design**	**Study** **population**	**Worksite health** **promotion programme**	**Participation** **level**	**Determinants** **of participation**	**OR [95%CI]**
Franklin 2006 [16]	cohort	Employees of an insurance company (n = 960)	Daily e-mail messages with links self-monitoring on nutrition and physical activity over 6 months.	40% (n = 388) (n = 345 completed baseline health survey)	male gender age (30–49) age (50+) white ethnicity married income, $30.000–$59.999 income, > $59.999	0.34 [0.24–0.49]* 1.30 [0.72–2.33] 1.47 [0.79–2.74] 1.22 [0.78–1.93] 1.43 [1.08–1.91]* 1.50 [1.08–2.09]* 0.90 [0.58–1.41]
Thomas 2006 [20]	cohort	Government employees (n = 3500)	1 information session with goal setting and subsequent pedometer use and e-mail support to increase physical activity over 4 weeks.	34% (n = 1195) (n = 927 provided demographic information)	male gender age (30–49) age (50+)	0.46 [0.39–0.54]* 0.73 [0.60–0.89]* 0.82 [0.66–1.02]
McCarty 2005 [19]	cohort	Employees of a health care system (n = 6539)	Self-monitoring and weekly e-mail support to increase physical activity and a healthy diet over a 16-week period	17% (n = 1129)	male gender	0.10 [0.08–0.14]*
Marshall 2003 [17]	RCT	University employees (n = 1409, results on n = 800 responded to questionnaire)	8 week programme with printed (I1) or website (I2) education and 4 reinforcement moments respectively by letter and e-mail.	46% (n = 655)	male gender age (yrs, mean) intermediate or high education married BMI (kg/m^2^, mean) good or excellent general health full-time employment academic job classification	0.77 [0.53–1.10] Δ = 0 yrs; d = 0.00 0.70 [0.46–1.07] 1.15 [0.78–1.70] Δ = 1 kg/m^2^; d = 0.14 0.69 [0.37–1.27] 0.69 [0.41–1.16] 0.79 [0.55–1.14]
Cornfeld 2002 [15]	cohort	Employees and spouses of 6 companies (n = 21396)	1-time health risk assessment with personalized feedback letters on cancer risk factors	21% (n = 4395)	male gender age (yrs, mean) Caucasian ethnicity	1.16 [1.09–1.24]* P: 44.8; all: 43.0 4.05 [3.52–4.67]*
Gold 2000 [21]	nonrandomized controlled trial	Employees of 6 organizations from the private and public sector (n = 1741)	Education materials, followed by 6-monthly telephone counselling sessions for 12 to 24 months on 7 risk areas (physical activity, nutrition, weight, smoking, stress management, back care, and cholesterol control)	35% (n = 607)	male gender age (yrs, mean) # health risks (lifestyle areas, 0–13)	1.13 [0.93–1.38] Δ = -1 yr Δ = -0.34 health risks*
Blake 1996 [14]	cohort community intervention trial	Employees in businesses participating in the Minnesota Heart Health Program intervention (n = 17626)	3 exercise competitions between companies with recording the type and minutes of daily exercise.	37% (n = 6495)	male gender company size, 45–500 employees company size, > 500 employees	0.28 [0.26–0.31]* 0.22 [0.19–0.25]* 0.09 [0.08–0.10]*
Hooper 1995 [22]	cross-sectional	University employees and spouses (n = 338)	Self-monitoring to increase physical activity over a period of 20 weeks.	30% (n = 103)	male gender higher education white ethnicity married full-time employment faculty employees	1.20 [0.70–2.07] 1.06 [0.66–1.71] 1.18 [0.45–3.11] 0.91 [0.50–1.66] 1.86 [1.01–3.43]* 0.68 [0.40–1.13]
Baer 1993 [13]	Nonrandomized controlled trial	Management-level male employees with elevated total cholesterol levels (n = 70)	An individual instruction, every 3 months group meetings, and monthly telephone support to decrease cholesterol level.	47% (n = 33)	age (yrs, mean) aerobic activity (days/wk, mean) cholesterol level > 6.17 weight (kg, mean) % body fat (mean) non smoker	Δ = 9 yrs*; d = 2.55 Δ = 0 days/wk; d = 0.00 14.3 [4.2–50.0]* Δ = 1 kg; d = 0.39 Δ = 1%; d = 0.24 3.00 [0.56–16.03]
Mavis 1992 [18]	cross-sectional	Stratified sample of university employees (n = 110 invited, 81% response)	Health fair and health habit modification programmes on exercise, weight control, stress management and smoking cessation.	25% of respondents (n = 22)	male gender age (yrs, mean) married/cohabiting income above $30.000 faculty employees (vs clerical/support)	0.30 [0.11–0.83]* Δ = 5.6* 1.89 [0.70–5.11] 0.62 [0.19–2.03] 0.11 [0.02–0.60]*

**Table 2:** Participation levels and determinants of participation in worksite health promotion programmes offering access to a fitness programme

**Study**	**Study design**	**Study population**	**Worksite health promotion programme**	**Participation level**	**Determinants of participation**	**OR [95%CI]**
Lechner 1997 [23]	cohort	Stratified sample of participants and non-participants from 3 companies (police force, chemical industry and banking) (n = 900, 98% response)	Fitness programme with supervised fitness exercises twice a week for 1 hour.	53% of stratified sample (n = 415)	male gender age (yrs, mean) # sick days (days, mean)	0.77 [0.53–1 .12] Δ = -1.1 yrs; d = -0.14 Δ = -1.93 days
Lewis 1996 [12]	cohort	Employees of a petrochemical R&D company	Fitness centre	fitness centre: 10% (n = 151)	male gender age, 31–50 age, 50+ higher education white ethnicity low fitness risk low obesity risk	0.53 [0.38–0.75]* 0.53 [0.35–0.79]* 0.43 [0.25–0.75]* 0.88 [0.56–1.37] 0.82 [0.54–1.23] 2.53 [1.52–4.21]* 1.67 [1.05–2.66]*
Heaney 1995 [26]	cohort	newly hired insurance company employees (n = 294)	Membership of a company's fitness centre within first year of employment.	19% (n = 55)	male gender age, 31–40 age, > 40 education some college education college graduate white ethnicity married pay grade 7–13 pay grade above 14 normal SBP normal DBP < 20% overweight 11–20% overweight 1–2×/wk physical activity > 2×/wk physical activity non smoker	2.04* 1.71 0.90 0.85 2.29* 0.66 0.90 4.29* 7.08* 0.86 1.75 1.06 1.05 0.85 1.04 1.37
Steinhardt 1992 [27]	cohort	Employees of an oil company (n = 2000) (76% of the participants (n = 400) and 88% of a random sample of non-participants (n = 246) completed the questionnaire)	Membership of a company's fitness centre within the first 6 months of existence	26% (n = 526)	*within questionnaire respondents:* male gender age, 30–49 age, 50+	0.89 [0.64–1.05] 0.66 [0.45–0.97]* 0.32 [0.18–0.56]*
Lynch 1990 [24]	cohort	Employees of an insurance company (n = 8069)	Membership of a company's fitness centre, within the first 2 yrs of existence.	28% (n = 2232)	male gender age men (yrs, mean) age women (yrs, mean) sick leave men (days, mean) sick leave women (days, mean)	1.62 [1.47–1.79]* Δ = -1.0 yrs* Δ = -5.3 yrs* Δ = -0.63 days* Δ = -0.93 days*
Shephard 1980 [25]	cross-sectional	Employees of a foods corporation (n = 2400) (76% of the participants (n = 409 and 44% of a random sample of non-participants (n = 374) completed the questionnaire)	Physical assessment and membership of the company's health fitness centre.	22% (n = 535)	male gender age, 30–49 age, 50+ activity past 3 months (mean), m activity past 3 months (mean), f health rating (mean) m health rating (mean) f	1.07 [0.89–1.30] 1.72 [1.37–2.17]* 1.14 [0.85–1.52] Δ = 0.16 Δ = 0.23 Δ = 0.12 Δ = 0.3*

**Table 3:** Participation levels and determinants of participation in multi-component worksite health promotion programmes

**Study**	**Study design**	**Study population**	**Worksite health promotion programme**	**Participation level**	**Determinants of participation**	**OR [95%CI]**
Stein 2000 [31]	cohort *(adjusted data)*	Benefit-eligible hospital employees (n = 2421)	Health risk assessment with results converted to dollar equivalents, plus a series of health promotion activities on physical activity, weight, nutrition, smoking, and stress management for variable time periods.	29%	male gender age 25–34 age 35–44 age 45–54 age 55+ white ethnicity not at risk (body fat) not at risk (cholesterol) full-time employment salary worker	0.38 [0.30–0.50]* 1.30 [1.03–1.62]* 1.43 [0.91–2.22] 1.79 [1.46–2.16]* 1.16 [1.13–1.17]* 1.28 [0.86–1.92] PR = 0.42 PR = 0.69 1.79 [1.41–2.22]* 1.54 [1.27–1.89]*
Lerman 1996 [33]	cohort	Career army personnel and spouses (n=not available)	A 4-day vacation programme with lectures, workshops, and access to sport facilities.	not available (n = 353)	male gender age 30–39 age, 40+ married intermediate education higher education non smoker	0.67* 1.66* 2.21* 4.14* 0.77 1.70* 4,81*
Lewis 1996 [12]	cohort	Employees of a petrochemical R&D company (n = 2290)	Health risk assessment, fitness centre, and education classes on physical activity, weight, nutrition, smoking, stress-management and blood pressure during a period of 2 yrs.	wellness programme: 64% (n = 1471)	male gender age, 31–50 age, 50+ higher education white ethnicity low fitness risk low nutrition risk low cholesterol risk low obesity risk low hypertension risk	0.34 [0.28–0.43]* 0.66 [0.51–0.85]* 0.57 [0.42–0.77]* 0.75 [0.59–0.96]* 0.97 [0.78–1.21] 1.45 [1.09–1.94]* 0.91 [0.56–1.50] 0.85 [0.66–1.09] 0.25 [0.15–0.43]* 0.41 [0.18–0.94]*
Sorensen 1996 [30]	cRCT *(adjusted data)*	Random sample of employees of intervention worksites in the WellWorksTrial (n = 2767)	Cancer-prevention intervention with several activities on individual and organizational level on nutrition, smoking, occupational safety for a 2-yr period.	nutrition programme: 49% (n = 1224)	male gender white collar worker vs. crafts/labourers	0.45 [0.36–0.56]* 1.52 [1.23–1.89]*
Knight 1994 [32]	cohort	University employees with 2 yrs of continuous employment (n = 4972)	Health screens and lifestyle improvement programmes on smoking cessation, weight control, stress management, nutrition education, fitness and blood pressure.	63% (n = 3122)	male gender age, 35–54 age, > 55 higher education white ethnicity	0.48 [0.42–0.54]* 0.96 [0.85–1.08] 0.64 [0.52–0.79]* 1.22 [1.09–1.37]* 1.12 [0.99–1.25]
Henritze 1992 [29]	cohort	Food Company employees (n = 1320)	Health screening followed by a variety of programmes during a 8-wk period: exercise equipment, and classes on activity, nutrition, hypertension and smoking.	52% (n = 692)	male gender age (yrs, mean) Caucasian ethnicity married shift work	0.57 [0.43–0.76]* P: 42.6 all workers: 43.0 0.83 [0.60–1.15] 1.13 [0.87–1.48] 0.57 [0.45–0.73]*
Brill 1991 [28]	cohort	Teachers in schools (n = 11830)	Health screen followed by 10-wk program with exercise sessions and health education classes.	33% (n = 3873)	male gender age, 36–50 age 50+ higher education white ethnicity	0.95 [0.86–1.04] 1.50 [1.37–1.64]* 1.34 [1.21–1.49]* 1.76 [1.56–2.00]* 2.04 [1.88–2.21]*

The demographic determinants most often reported were sex (n = 22), age (n = 19), ethnicity (n = 10), education (n = 8), marital status (n = 7), and income (n = 3) (Tables 1, 2, 3). Most studies reported a higher participation among women (n = 16), of which 12 reached statistical significance [12,14,16,18-20,29-33]. In contrast, 6 studies found a higher participation among men [15,21,22,24-26], of which 3 were statistically significant [15,24,26]. A higher participation among female employees was found for educational and multi-component programmes, but not for fitness centre facilities (Table 2).

Contradictory results were reported for age with both statistically significant higher by [13,18,28,31,33] and lower [12,20,24,27,32] participation levels among older employees. For marital status, five [16-18,29,33] out of seven studies found a higher participation level among married or cohabiting employees (of which two were statistically significant [16,33]). Two out of six studies that reported a higher participation level among Caucasian or white employees found a statistically significant difference in comparison with black or Hispanic employees [15,28]. None of the four studies reporting a lower participation among Caucasian or white employees reached statistical significance [12,26,29]. Concerning education and income, both positive and negative associations were reported. Four positive statistically significant associations were found for a higher education level [26,28,32,33], and one study reported a higher participation level for those with a lower education level [12]. One out of three studies showed a higher participation level among workers with a higher income [26].

A large variety of health-related determinants were addressed, most notably (over)weight (n = 6), physical activity level (n = 5), smoking (n = 3), cholesterol level (n = 3), general health/health risks (n = 3), blood pressure (n = 2), and nutrition (n = 1). For health-related determinants, there is no consistent evidence for a higher participation among healthier workers. Lewis (1996) reported contrary findings for the multi-component and fitness centre programme: a higher participation among employees with obesity and hypertension risk in the multi-component programme and a higher participation among those with a low fitness and obesity risk in the fitness centre intervention [12]. One study reported a higher participation those with an elevated cholesterol level in a nutrition programme [13]. Some studies reported a higher participation level among those with less health risks [21,25], and those with less sick leave [24].

Work-related determinants studied were job type (n = 5), employment (full/part-time) (n = 3), company size (n = 1), and work shift (n = 1). The only statistically significant associations were a higher participation among white-collar or workers with secure contracts [30,31], fulltime-workers [22,31], and employees in smaller companies [14]. A lower participation level was found for those with shift work [29].

In Table 4 the pooled ORs for the demographic determinants are provided. In accordance with the individual studies described above, a statistically significantly higher participation level among female workers was found (OR = 1.67, 95%CI: 1.25–2.27). After stratifying by programme type, no difference between male and female workers was observed in the fitness centre studies (OR = 1.02, 95%CI: 0.68–1.53) as compared to education/counselling and multi-component studies (OR = 2.00, 95%CI: 1.43–2.78). A significant higher participation level was found for married/cohabiting workers compared to other (OR = 1.25, 95% CI: 1.05–1.48). Age, education, and income had no effect on participation.

**Table 4:** Pooled odds ratios and corresponding 95% confidence intervals for participation levels for specific demographic determinants

**determinant**		studies (n)*	**Pooled OR [95%CI]**	
sex	(female:male)	20	**1.67**	**[1.25–2.27]**
				
age	(middle:young)	8	0.93	[0.71–1.24]
age	(old:young)	8	0.76	[0.54–1.06]
				
education	(moderate/high:low)	6	1.04	[0.77–1.40]
income	(high:low)	2	0.86	[0.56–1.31]
				
ethnicity	(white:other)	9	1.33	[0.91–1.95]
				
marital status	(married:other)	5	1.25	[1.05–1.48]

* The total number of studies included in this table varies per characteristic. For each demographic characteristic, only studies enabling to calculate OR's and CI's are included.

Table 5 shows higher participation levels in programmes offering incentives, and in multi-component interventions. No difference in participation levels was found between programmes requiring a fee and programmes with free participation. The difference in mean participation level between studies aimed at physical activity and studies aimed at multiple behaviours reached statistical significance.

**Table 5:** Pooled participation levels and corresponding 95% confidence intervals for study characteristics

**study characteristics**	number of studies (n)*	**number of participants (n)**	**mean (%) [95% CI]**	
incentive	9	11960	33.5%	[33.3% – 33.8%]
no incentive	13	18060	30.7%	[30.5% – 30.9%]
				
fee	4	4053	32.2%	[31.8% – 32.7%]
no fee	18	26740	31.7%	[31.5% – 31.9%]
				
education/counselling	10	15022	28.0%	[27.8% – 28.2%]
fitness	6	3914	25.8%	[25.4% – 26.1%]
multi-component	6	11084	43.3%	[42.9% – 43.3%]
				
physical activity	10	6474	29.2%	[28.9% – 29.5%]
multiple behaviours	12	23546	32.6%	[32.4% – 32.8%]

## Discussion

In this systematic review, participation levels in health promotion interventions at the workplace were typically below 50%. A large variation in participation levels and determinants of initial participation in worksite health promotion was shown, and except for sex few statistically significant associations with initial participation were found. Female workers had a higher participation than men, but this difference was not observed for interventions consisting of fitness centre programmes. In addition, the review showed that programs that provide (1) incentives, (2) offer a multi-component strategy, (3) focus on multiple behaviours rather than on physical activity only have a higher overall participation level.

A major reason for choosing the worksite as setting for health promotion is the possibility to reach large groups [7,9]. It is striking that the differences between participation levels were large, with mainly low participation levels, but also levels up to 64%. The large variation is comparable to the findings of Glasgow and colleagues (1993), who found participation levels ranging from 20% to 76%. The authors noticed that attending a single screening does not require much commitment [9]. In our review, we included only studies evaluating interventions aimed at physical activity and/or nutrition, and therefore excluded studies evaluating only a single health risk assessment (HRA). The median participation level found in a review on 24 studies by Bull and colleagues (2003) was higher than the median reported in this review (61% versus 34%) [7]. It is not clear if Bull and colleagues included studies evaluating a HRA.

The findings on determinants of participation are in accordance with the review of Glasgow and colleagues [9]. The overall view is that female employees are more likely to participate in health promotion programmes than male employees.

After pooling, an overall higher participation level for married employees was found. All other demographic characteristics showed no consistent pattern. Only for age, there appeared to be a trend with a higher participation among younger employees, and lowest participation level among the oldest age group. As mentioned, just few statistically significant associations for health- and work-related determinants were found. Several studies have reported higher participation in smaller worksites albeit without providing quantitative information [34,35]. This finding is supported in this review by the included study of Blake and colleagues (1996) [14]. No pooled ORs were calculated for the health- and work-related determinants due to the large variation in definition of determinants and programmes evaluated.

More than 80% of the studies evaluating a WHPP on nutrition or PA did not report any determinants of non-participants. In 1993, Glasgow and colleagues already recommended that future studies should report participation levels, the number of employees entering the programme, and demographic information [9]. This information is needed to gain insight in potentially selective participation and external validity. Just few studies included information on educational level and income. Since unhealthy lifestyles are more common among lower socio-economic groups, it is important to get insight in the reach (and effectiveness) in these specific groups. Information on determinants should be an essential aspect of a process evaluation. In the RE-AIM framework for the evaluation of the public health impact of health promotion interventions, the 'reach' dimension is included which is measured by comparing records of participants and complete sample information for a defined population, in this case the worksite [36]. In the recent CONSORT statements it is emphasized to include information on the eligible participants in order to increase the validity [37].

In total, 64 out of 130 (49%) associations between determinants and participation did not reach statistical significance. These null associations may be the result of a small sample size and lack of statistical power, and the presence of another risk factor or confounder [38]. It is not likely that most null associations are explained by the sample size or confounding, because most studies had sample sizes larger than 500 subjects, and most ORs were calculated by means of univariate analysis. Thus, the lack of a clear health-related selection in participation suggests that WHPPs are able to reach those most-at-risk and, hence, provide a valuable setting.

After stratification of the demographic determinants by programme type, it appeared that fitness centre studies do not suffer from a lower participation among men. Further, no statistically significant differences in demographic determinants were found between programme categories. The finding that fitness centre studies do not favour female workers in comparison with other programme categories, suggests that the content of intervention programmes should be tailored to the population characteristics.

In addition to determinants that may play a role in the uptake of interventions in the context of work settings, several programme characteristics were associated with participation. First, this review and others [39] suggest that the inclusion of an incentive can have beneficial effects on reach, hence increasing the absolute number of people who engage in health-related activities. Second, the present finding that more multi-component interventions do not decrease the uptake is in itself reassuring. A potential explanation for this finding may be that these interventions offer a large choice for potential participants. It could be hypothesized that multi-component interventions may have bigger participation levels as it matches with a larger array of people, whereas a mismatch is more likely for single components whereby persons may not see the need or be ready to engage in a particular activity. Finally, in this review a fee for participation was not identified as a barrier to participate. The 4 studies reporting on interventions with a fee for participation included 1 very large study [28]. Excluding this study showed among the remaining 3 studies a lower participation level (participation level: 24.3%; 95% CI: 22.7%–25.8%) as compared to studies not requiring a fee for participation (participation level: 31.7%; 95% CI: 31.5–31.9%). This indicates that the results of the pooled analysis should be interpreted carefully depending on the studies included.

Low participation levels will result in decreased (cost-)effectiveness of intervention programmes on population level and a potentially decreased generalizability of the results [40]. Implications for raising participation levels in WHPPs are the provision of incentives, or a broad array of programme offers. To what degree these strategies affect also compliance to an intervention programme should be considered.

### Limitations

This systematic review has some limitations. First, the literature search was limited to two electronic databases, with an overlap of 86% of the articles. With just two electronic databases and only English publications included, it is possible that we missed some useful studies. We assume this does not have a major effect on the findings. Second, many interventions are conducted in practice that are not well-evaluated and not published in scientific literature. This review is limited to the published research. Third, 8 out of 30 studies were excluded because they reported only qualitative information on initial participation. Fourth, pooling of all determinants was impossible because of the large heterogeneity in definition of initial participation, in programme components, and measurement of determinants. Finally, due to the limited information provided in studies, the possibility to study the interaction between determinants and programme characteristics was restricted.

## Conclusion

In this systematic review, participation levels in health promotion interventions at the workplace were typically below 50%. This will greatly influence the effects of these interventions. Few studies evaluated the influence of health, lifestyle and work-related factors on participation, which hampers the insight in the underlying determinants of initial participation in worksite health promotion. This insight is essential to develop tailored intervention programmes, to reach those who need it most, and to increase generalizability across all workers.

## Competing interests

The authors declare that they have no competing interests.

## Authors' contributions

SR carried out the design, literature search, data extraction, data-analysis and drafted the manuscript. FvL and PvE participated in discussing the paper, providing methodological input, and helped to draft the manuscript. AB conceived the study, and participated in its design, data-extraction and coordination and helped to draft the manuscript. All authors read and approved the final manuscript.
